# Case Report: Is Pneumoperitoneum the Only Indication for Surgery in Necrotizing Enterocolitis?

**DOI:** 10.3389/fped.2021.714540

**Published:** 2021-08-06

**Authors:** Ying Wei, Yanhong Zhu, Xiaoping Luo, Ling Chen, Xiaolin Hu

**Affiliations:** Department of Pediatrics, Tongji Hospital, Tongji Medical College, Huazhong University of Science and Technology, Wuhan, China

**Keywords:** necrotizing enterocolitis, neonate, pneumoperitoneum, surgery, treatment

## Abstract

**Objective:** This study aims to explore whether pneumoperitoneum is the only surgical indication for neonates with necrotizing enterocolitis (NEC) and to analyze when early surgical intervention should be considered.

**Methods:** A retrospective case series study was conducted including six neonates with stage 2a−2b NEC who received surgeries without absolute indication but with failed conservative treatment. In the meantime, seven infants who received surgery due to pneumoperitoneum and 32 infants treated with conservative treatment were also included for comparison.

**Results:** Our results indicated that the six infants who received surgical treatment without pneumoperitoneum had a better prognosis compared to the seven infants who underwent surgical treatment after the onset of pneumoperitoneum. None of the infants who received early surgical treatment developed short bowel syndrome or neurodevelopmental impairment, while four out of six infants exhibited growth retardation. On the other hand, a total of five out of the seven infants who received surgical treatment after pneumoperitoneum forfeited further treatment, two developed short bowel syndrome, and one experienced neurodevelopmental impairment. Lower gestational age and birth weight, fetal growth restriction (FGR), perinatal asphyxia, postnatal steroid and vascular active drug use, blood transfusion, and hemodynamic significant patent ductus arteriosus were identified as risk factors associated with surgical repair for infants with NEC. In a laboratory test, infants who needed surgeries had a lower platelet count and a higher C-reactive protein value.

**Conclusion:** Aggressive surgical treatment should be considered in infants with severe necrotizing enterocolitis before the onset of pneumoperitoneum. Lower gestational age and birth weight, FGR, perinatal asphyxia, postnatal steroid and vascular active drug use, blood transfusion, and hsPDA may be associated with surgical repair for infants with NEC.

## Introduction

Necrotizing enterocolitis (NEC) is one of the most common life-threatening diseases affecting the gastrointestinal tract of premature infants and newborns (1). The incidence of NEC had been reported as 1–3%0 in live births, which increases to 7% in premature infants <1,500 g and to 14% in extremely low birth weight (ELBW) infants between 400 and 750 g (1). It has been estimated that a total of 30–40% of all infants who were diagnosed with NEC eventually need surgical interventions (2–4). The overall mortality rate in surgical NEC was 20–30% and up to 50% in ELBW infants (5).

Pneumoperitoneum is the only absolute indication for surgery in NEC patients. However, studies had demonstrated that pneumoperitoneum only presented in less than half of NEC infants who eventually need surgical treatment (6), which meant opinions on the criteria of surgery in NEC patients were controversial. However, a growing number of researchers had also proposed some relative indications for surgical NEC, including clinical or radiological findings such as increased abdominal circumference over time, marked erythmia of the abdominal wall, and fixed abdominal masses as well as worsening apnea and bradycardia, temperature instability, and hypotension despite maximized medical therapy (7), but there has been a lot of debate about these relative surgical indications, without a definitive conclusion. However, in our experience, we performed surgery in NEC infants who had progressive deteriorated disease, recurrent NEC, or feeding intolerance post-NEC despite conservative treatment. It was defined as an aggressive surgical treatment since none of the infants truly had perforation in the radiology.

In this study, we retrospectively examined the prognosis of infants who received aggressive surgical treatment without the onset of perforation and pneumoperitoneum as identified by imaging. The clinical characteristics, surgical results, and follow-up outcomes after surgery were collected and compared to NEC infants treated with conservative management in the same period to identify the risk factors attributed to the surgical requirements. We also compared the aggressive surgical group with patients who received surgery only after the onset of pneumoperitoneum secondary to intestinal perforation to explore the best timing to perform surgery in NEC.

## Methods

In this retrospective case series study, the medical records of neonates admitted to Tongji Hospital of Huazhong University of Science and Technology from January 2016 to December 2019 were retrospectively reviewed. We included infants clinically diagnosed with NEC with a gestational age of <32 weeks or a birth weight of <1,500 g, excluding infants with severe congenital malformation or congenital digestive tract malformation or who died or withdrew from treatment during hospitalization due to other factors except NEC. The signs and symptoms for NEC were nonspecific and included abdominal distention, abdominal wall cellulitis, feeding intolerance, emesis, bloody stools, temperature instability, lethargy, bradycardia, apnea, and hypotension. The biochemical indicators supporting a diagnosis of NEC included acidosis, thrombocytopenia, and neutropenia (7). The diagnosis and stratification of NEC was referenced to the Bell staging criteria, which was firstly devised in 1978 and modified by Walsh and Kliegman in 1986 (8, 9).

Clinical data such as maternal conditions, neonatal general information including gestational age and birth weight, perinatal status, presence of hemodynamic significant patent ductus arteriosus (hsPDA), and antibiotic treatment were collected. No consensus on clinical or sonographic criteria that could define hsPDA had been reported. We entitled a hsPDA as the PDA that required medical or surgical treatment, which entailed using various parameters to identify whether it had a high likelihood of remaining open or presented with adverse hemodynamic effects. Several echocardiographic variables had been used to assess the significance of a PDA: ductal diameter≥2 to 3 mm or ductal diameter ≥ mean pulmonary artery, a left atrium-to-aortic root diameter ratio ≥1.4, left ventricular enlargement, left ventricular ejection fraction <50%, *etc*. The clinical parameters included the need for vasopressors/inotropes, the need for ventilatory support and pulmonary edema, feeding intolerance, rising creatinine, etc. (10, 11). Ultrasonography was performed by a sonographer specializing in cardiac ultrasound and with 10 years of working experience in the Department of Ultrasonography in our hospital. Data on disease presentation, laboratory findings at the onset of disease, as well as surgical parameters were also collected. All patients were regularly followed up in the High-Risk Infant Follow-up program of our hospital. Growth and development status as well as postoperative complications were examined during the follow-up. Infants who underwent surgery due to pneumoperitoneum as the absolute indication (seven patients) and those who only received conservative treatment (32 patients) were also included in this study for comparison. This study was approved by the ethics committee of Tongji Hospital, Tongji Medical College of Huazhong University of Science and Technology.

## Result

Eventually, a total of 45 NEC patients in the time period were included in our study, among whom six neonates received surgical treatment without absolute indication after a failed conservative treatment. The medical history, disease manifestations, detailed laboratory findings, and follow-up information of all six patients who received surgical treatment without pneumoperitoneum are summarized in Table 1. Their clinical courses were described as follows:

**Table 1:** Clinical data and follow-up information for six infants with necrotizing enterocolitis (NEC) who underwent early surgery.

	**Case 1**	**Case 2**	**Case 3**	**Case 4**	**Case 5**	**Case 6**
General information						
Gestational age (weeks)	24 w	28 w + 5	26 w	29 w + 1	27 w + 1	31 w
Birth weight (gram, percentile)	570 (35%)	1,440 (91%)	780 (47%)	1,330 (63%)	950 (46%)	1,160 (11%)
Sex	Female	Female	Female	Male	Male	Male
First meconium (days)	Seven	One	Two	One	One	Two
Feeding intolerance before NEC	Yes	Yes	No	Yes	No	Yes
Enteral nutrition	Mixed feeding	EBM	EBM	EBM	EBM	Preterm formula
Days of disease onset (days, CGA)	31 (28 w + 3)	16 (31 w)	43 (32 w + 1)	12 (30 w + 6)	24 (30 w + 4)	20 (33 w + 6)
Maternal information						
Maternal age at delivery (years)	30	30	40	29	41	31
Reason for preterm birth	Maternal placental abruption	Maternal placental abruption	Maternal placental abruption	TTTS, fetal distress	Maternal severe preeclampsia	Maternal severe preeclampsia
Maternal complications	No	No	No	GDM	GDM, PIH	GDM, PIH
Risk factors						
Fetal growth retardation	No	No	Yes	No	Yes	Yes
Asphyxia at birth needing NRP	Yes	Yes	Yes	Yes	Yes	No
Use of steroid before NEC	No	No	Yes	Yes	Yes	Yes
Use of inotropes before NEC	Yes	No	Yes	No	Yes	Yes
Use of antibiotics before NEC	Yes	No	Yes	Yes	Yes	Yes
Blood transfusion before NEC	Yes	Yes	Yes	No	No	No
MV before NEC	Yes	Yes	Yes	Yes	Yes	Yes
hsPDA needing treatment	Yes	No	Yes	Yes	Yes	Yes
Gastrointestinal manifestations	Abdominal distention, large volume residues, emesis, hematochezia, hypoactive bowel sounds	Abdominal distention, large volume residues, hematochezia, rarely defecate autonomously, hypoactive bowel sounds	Abdominal distention, large volume residues, hematochezia tenderness, hypoactive bowel sounds	Abdominal distention, large volume residues, emesis, hematochezia, hypoactive bowel sounds	Abdominal distention, tenderness, large volume residues, hematochezia, hypoactive bowel sounds	Abdominal distention, large volume residues, emesis, hematochezia, hypoactive bowel sounds
Systemic symptoms	Temperature instability, frequent apnea, desaturation	Temperature instability, desaturation	Frequent apnea desaturation, and tachypnea	Apnea, desaturation, piebaldskin	Temperature instability, tachypnea, ochrodermia, desaturation	Temperature instability, desaturation
The blood test						
WBC (×10^9^/L)	7.93	9.22	3.6	11.1	15.19	7.49
Neu (×10^9^/L)	2.94	2.28	2.1	3.35	10.09	3.3
PLT (×10^9^/L)	488	602	45	158	80	153
Highest CRP (mg/L)	30	0.5	118	2.4	118.6	57.66
Na (mmol/l)	135.4	133.8	133.4	142.4	132.8	138.4
Blood culture	Negative	Negative	Negative	Negative	Negative	Negative
Abdominal X-ray	Extensive intestinal dilatation	Extensive intestinal dilatation	Extensive intestinal dilatation	Extensive intestinal dilatation	Extensive intestinal dilatation	Extensive intestinal dilatation
Abdominal ultrasound	Intestinal pneumatosis	NA	Intestinal wall thickened, intestinal loop fixed, ascites	Intestinal pneumatosis	NA	NA
NEC stages^a^ before surgery	2a	2a	2b	2b	2b	2a
Conservative treatment	At DOL 31, NPO for 10 days, at DOL 71, NPO for 2 weeks	At DOL 16, NPO for 4 days, at DOL 33, NPO for 12 days	Abdominal symptoms continued to worsen during fasting	At DOL 12, NPO for 14 days, at DOL 30, NPO for 6 days	Abdominal symptoms continued to worsen during fasting	At DOL 20, NPO for 14 days and still worsened
Reason for surgery	Recurrent feeding intolerance	Recurrent feeding intolerance	Worsened abdominal sign	Recurrent feeding intolerance	Worsened abdominal sign	Recurrent feeding intolerance and worsened abdominal sign
Operation time (CGA)	101 days (38 w + 3)	45 days (35 w + 1)	46 days (32 w + 4)	35 days (34 w + 1)	27 days (31 w)	38 days (36 w + 3)
Postoperative PN (days)	18	17	18	21	30	30
Operation method	Exploratory laparotomy and ileostomy	Exploratory laparotomy and ileostomy	Exploratory laparotomy and ileostomy	Exploratory laparotomy and ileostomy	Exploratory laparotomy and ileostomy	Exploratory laparotomy, segmentectomy for necrotic bowel, end-to-end intestinal anastomosis
Intraoperative diagnosis	Intestinal stenosis, ascites,NEC	Ascites, intestine dilatation, colon pneumatosis, NEC	NEC, colon perforation, diffuse peritonitis	Pneumatosis, intestinal dilatation, NEC	Ileocecal necrosis, NEC	Intestine dilatation, rigid moveless intestinal wall, NEC, ascites
PNAC	Yes	No	No	Yes	No	Yes
Total hospitalization days (CGA)	123 days (41 w + 4)	67 days (38 w + 2)	95 days (39 w + 4)	58 days (37 w + 3)	63 days (36 w + 1)	97 days (44 w + 6)
Follow-up information						
Time of closing the stoma (CA)	16 m (12 m)	16 m (13 m)	14 m (11 m)	11 m (8 m)	8 m (5 m)	NA
Height at last follow-up (%)	88 cm (90.0%)	83 cm (73.8%)	86 cm (53.4%)	71 cm (47.1%)	93 cm (20.7%)	95 cm (5.4%)
Weight at last follow-up (%)	8.5 kg (5.0%)	9.5 kg (25.0%)	11 kg (41.7%)	8.5 kg (50.0%)	12 kg (7.2%)	15 kg (33.3%)
Short bowel syndrome	No	No	No	No	No	No
Neurodevelopmental impairment	No	No	No	No	No	No

*GDM, gestational diabetes mellitus; PIH, pregnancy-induced hypertension; NEC, necrotizing enterocolitis; CGA, correct gestational age; MV, mechanical ventilation (including invasive and non-invasive); EBM, exclusive breast milk; FGR, fetal growth restriction; TTTS, twin-to-twin transfusion syndrome; WBC, white blood cell; Neu, neutrophil; PLT, platelet; Na, blood natrium; CRP, C-reactive protein; DOL, day of life; NRP, neonatal resuscitation program; NEC, necrotizing enterocolitis; PN, parenteral nutrition; PNAC, parenteral nutrition associated cholestasis; CA, corrected age; hsPDA, hemodynamic significant patent ductus arteriosus; NA, not available*.

a *NEC stratification was referenced to the Bell staging criteria, which was first devised in 1978 and modified by Walsh and Kliegman in 1986*.

Case 1 had a gestational age of 24 weeks and a birth weight of 570 g. The main cause of premature birth was premature rupture of membrane and inevitable abortion. The baby had no breath at birth and was resuscitated by neonatal resuscitation program, including intubation and chest compression. The mother was 30 years old and had no pregnancy complications. The baby had a marked delayed meconium excretion, which initiated at approximately 1 week of age, despite the use of glycerin enema. She was mainly fed with expressed breast milk and had at least two significant feeding intolerances prior to the onset of NEC. The risk factors associated with NEC were a history of asphyxia, hsPDA requiring medical treatment (ibuprofen), prior use of mechanical ventilation, vasoactive drugs, and antibiotics, and a prior history of blood transfusion. At 31 days of life (28 w + 3 of postmenstrual age), the patient developed large-volume residuals, abdominal distention, emesis, hypoactive bowl sounds, *etc*., when breastfed at 56 ml/kg per day. The systemic symptoms included temperature instability, frequent apnea, and oxygen desaturation. The abdominal X-ray and ultrasound both showed extensive dilatation of the intestine and gas in the intestinal wall. Therefore, the infant was diagnosed with NEC IIa and received intestinal rest, antibiotics, parenteral nutrition, and respiratory support treatment. After 10 days of NPO, feeding was restarted. At 71 days of age (31 w + 1 of postmenstrual age), the above-mentioned symptoms presented again at 87.6 ml/kg/day of preterm formula. Intestinal resting was then started for another 2 weeks in addition to other conservative treatments, yet the symptoms persisted with a bloody stool, marked abdominal distention, and significantly weakened bowel sounds. No significant abnormalities were found in complete blood count and blood culture, while the C-reactive protein (CRP) was up to 30 mg/L. Exploratory laparotomy and ileostomy were performed at 101 days of age (38 w + 3 of postmenstrual age), and the intraoperative diagnosis was incomplete small intestine obstruction, ascites, and necrotizing enterocolitis. Receiving total intestinal feeding at 18 days after surgery, the patient was discharged at 123 days (41 w + 4 postmenstrual age) and came to our hospital again to close the stoma at the age of 1 year and 4 months. The patient was regularly followed up in high-risk follow-up clinics. At the corrected age of 18 months, the child was 88 cm in height (90th percentile) and 8.5 kg in weight (5th percentile), without signs of short bowel syndrome. The Gesell scores showed that the gross motor, fine motor, language, and social adjustment abilities were all consistent with the corrected age.

Cases 2–6 are summarized in Table 1. Similar to case 1, cases 2 and 4 also had repeated feeding intolerance that worsened with time, which eventually led to surgery. In cases 3, 5, and 6, the condition of the infants deteriorated rapidly, and they developed a series of sepsis-like symptoms within only 3 days, despite of NPO, antibiotics, and supportive treatment. None of these infants had signs of pneumoperitoneum in radiology. However, an operation was arranged due to the severe progression being irresponsive to medical treatment. Intraoperatively, cases 2–6 were found to have varied range of intestinal necrosis, ascites, intestinal dilation, and intestinal wall gas accumulation, whereas case 3 showed intestinal perforation and diffuse peritonitis. Eventually, cases 2–5 underwent exploratory laparotomy and ileostomy, while case 6 underwent exploratory laparotomy, segmentectomy for necrotic bowel, and end-to-end intestinal anastomosis.

Table 2 shows a comparative analysis of clinical data, risk factors, and laboratory tests between patients who received aggressive surgical treatment and those who were managed only by conservative treatment. Compared to the conservative treatment group, infants who received aggressive surgery had lower mean gestational age (27 weeks and 5 days) and birth weight (1,038.33 g), higher proportions of fetal growth restriction (FGR; 50.0 vs. 18.8%), and a higher incidence of perinatal asphyxia (83.3 vs. 46.9%) as well as more frequent usage of postnatal steroids (33.3 vs. 12.5%) and vascular active drug use (66.7 vs. 18.8%) before the onset of NEC. In addition, the blood transfusion rate (50.0 vs. 21.9%) and the occurrence of hsPDA (83.3 vs. 31.3%) were also higher in patients who received aggressive surgery. In the laboratory test, the mean platelet count was lower (254.33 vs. 305.44 × 10^9^/L), and the mean CRP value was higher (54.53 vs. 22.74 mg/l) in the aggressive surgical group. The parenteral nutrition (PN) duration after disease onset (46.83 vs. 24.97 days), total PN duration (71.17 vs. 46.47 days), and total hospitalization days (83.83 vs. 55.06 days) were significantly longer, and the incidence of parenteral nutrition-associated cholestasis (50 vs. 28.1%) was higher in the aggressive surgical group compared to those in the conservative treatment group.

**Table 2:** Comparative analysis of clinical data, risk factors, and laboratory tests between the early surgery group and the conservative treatment group.

	**Medical NEC** **(** ***N*** **= 32)**	**Early surgical NEC** **(** ***N*** **= 6)**
GA, weeks [median (min–max)]	30 W + 3 (25 + 6–34 weeks)	27 W + 5 (24–31 weeks)
Birth weight [median (min–max)]	1,387.81 g (900–1,870 g)	1,038.33 g (570–1,440 g)
Age at onset, days [median (min–max)]	21.47 (7–50)	24.33 (12–43)
FGR, *N* (%)	6 (18.8%)	3 (50.0%)
Presence of asphyxia, *N* (%)	15 (46.9%)	5 (83.3%)
Use of steroid, *N* (%)	4 (12.5%)	2 (33.3%)
Use of inotropes, *N* (%)	6 (18.8%)	4 (66.7%)
Use of antibiotics before NEC, *N* (%)	23 (71.9%)	5 (83.3%)
Blood transfusion before NEC, *N* (%)	7 (21.9%)	3 (50.0%)
Formula or mixed feeding, *N* (%)	15 (46.9%)	2 (33.3%)
hsPDA, *N* (%)	10 (31.3%)	5 (83.3%)
Mechanical ventilation, *N* (%)	28 (87.5%)	6 (100.0%)
Maternal factors		
Maternal age at delivery (years)	31.1 (22–48)	33.5 (29–41)
Cesarean delivery, *N* (%)	24 (75.0%)	5 (83.3%)
Multiple births, *N* (%)	12 (37.5%)	3 (50.0%)
PIH, *N* (%)	11 (34.4%)	2 (33.3%)
GDM, *N* (%)	8 (25.0%)	3 (50.0%)
Test results		
WBC (×10^9^/L), (min, max)	9.75 (3.17, 20.21)	9.09 (3.60, 15.19)
N (×10^9^/L), (min, max)	4.30 (0.48, 12.59)	4.01 (2.10, 10.09)
PLT (×10^9^/L), (min, max)	305.44 (68, 584)	254.33 (45, 602)
CRP (mg/L), (min, max)	22.74 (0.5,116.8)	54.53 (0.5,118.6)
Na (mmol/l), (min, max)	136.94 (127.7, 145.8)	136.03 (132.8, 142.4)
Positive blood culture, *N* (%)	2 (6.3%)	0 (0%)
PN after disease onset (days) (min, max)	24.97 (8, 75)	46.83 (21, 88)
Total PN (days) (min, max)	46.47 (17, 96)	71.17 (56, 119)
Total hospitalization days (days) (min, max)	55.06 (27, 100)	83.83 (58, 123)
PNAC, *N* (%)	9 (28.1)	3 (50)

*GDM, gestational diabetes mellitus; PIH, pregnancy-induced hypertension; NEC, necrotizing enterocolitis; GA, gestational age; MV, mechanical ventilation (including invasive and non-invasive); FGR, intrauterine growth restriction; WBC, white blood cell; Neu, neutrophil; PLT, platelet; Na, blood natrium; CRP, C-reactive protein; hsPDA, hemodynamic significant patent ductus arteriosus; PN, parenteral nutrition*.

Table 3 shows the comparative analysis between infants receiving surgery with and without an image-identified pneumoperitoneum. By comparison, the mean platelet count of seven infants in the absolute operation group was lower (158.17 vs. 254.33 × 10^9^/L) and the mean CRP was higher (114.28 vs. 54.53 mg/l). The ratio of positive blood culture was also higher than those without pneumoperitoneum but received aggressive surgical treatment (71.4 vs. 0%). In terms of prognosis, five out of seven infants with perforation were forfeited with further treatment and died due to a serious condition such as total intestinal necrosis. Among the only two surviving infants in the absolute surgery group, two had growth failure, two had short bowel syndrome, and one had neurodevelopmental impairment. By contrast, infants in the group without pneumoperitoneum were all discharged safely. Four of them had growth retardation at the follow-up of 18 months. None developed short bowel syndrome or neurodevelopmental impairment.

**Table 3:** Comparative analysis of general data and prognosis between the early and absolute surgery groups.

	**Early surgical necrotizing enterocolitis (NEC)** **(** ***N*** **= 6)**	**Absolute surgical NEC** **(** ***N*** **= 7)**
Gestational age, weeks [median (min–max)]	27 W + 5 (24–31 weeks)	28 W + 4 (25 + 1–31 + 2 weeks)
Birth weight [median (min–max)]	1,038.33 g (570–1,440 g)	1,218.6 g (950–1,630 g)
Age at onset, days [median (min–max)]	24.33 (12–43)	26.29 (12–46)
Test results		
WBC (×10^9^/L), (min, max)	9.09 (3.60–15.19)	9.11 (2.83–17.00)
N (×10^9^/L), (min, max)	4.01 (2.10–10.09)	5.47 (1.45–13.20)
PLT (×10^9^/L), (min, max)	254.33 (45–602)	158.17 (82.0–371.0)
CRP (mg/L), (min, max)	54.53 (0.5–118.6)	114.28 (42.6–185.2)
Na (mmol/l), (min, max)	136.03 (132.8–142.4)	131.49 (123.8–137.7)
Positive blood culture, *N* (%)	0/6 (0%)	5/7 (71.4%)
Status of discharge		
Forfeited and died/improved and discharged	0/6	5/2
Follow-up information		
Mean follow-up age	2 years and 3 months (11 months−3 years and 9 months)	2 years and 6 months (1 year and 8 months−3 years and 5 months)
Short bowel syndrome	0/6	2/2
Growth failure	4/6	2/2
Neurodevelopmental impairment	0/6	1/2

*WBC, white blood cell; N, neutrophil; PLT, platelet; Na, blood natrium; CRP, C-reactive protein*.

## Discussion

### What Is the Effect of Aggressive Surgical Strategy in Infants With NEC?

NEC is the most common life-threatening condition affecting the gastrointestinal tract in premature infants and newborns (1). According to the guidelines, the identification of pneumoperitoneum on imaging was the only absolute criterion for surgery. However, the fact that less than half of the surgical patients met the absolute criterion indicated that a considerable number of surgery decisions were made not solely on the absolute indication.

Clinicians insisting strictly on the absolute criterion believed that patients who received a surgical treatment of NEC were at an increased risk of morbidity and mortality, with long-term complications including short bowel syndrome, growth failure, and neurodevelopmental impairment (7). However, some other clinicians argued that NEC should have relative indications. It seemed that severe NEC rather than the surgery itself should be responsible for the poor outcomes. A surgery performed earlier rather than in the later course of the disease may be beneficial. Therefore, some experts may consider aggressive surgery especially for patients who had not yet developed signs of pneumoperitoneum or localized abscess (12).

In this study, five out of seven infants who met the absolute surgical indication eventually died. The survivors developed sequelae such as growth retardation, short bowel syndrome or intestinal failure, and neurodevelopmental impairment. The findings were consistent with previous research on patients with a surgical treatment of NEC, which proved that performing surgery isolated on the absolute criterion seemed not optimal for infants with a severe disease. On the contrary, our data indicated that all the six infants receiving an aggressive operation improved rapidly and recovered well at discharge. Intestinal failure and neurodevelopment impairment were not observed among these patients in the follow-up assessment. Therefore, the clinical outcomes of the aggressive operation group were significantly better than those with an absolute indication. The results indicated that aggressive surgical intervention should be considered in infants with NEC and who were refractory to conservative treatment before the onset of pneumoperitoneum.

Compared with the conservative treatment group, the aggressive surgical group had a significantly longer duration of PN and hospitalization after the onset of NEC. It may be related to the few surgical cases, which were characterized by either recurrent feeding intolerance requiring elongated PN or more severe clinical presentations. However, the infants in the aggressive surgical group were re-fed smoothly post-operation. They reached full feeding in 17–30 days after surgery, which was comparable to the time required by the conservative treatment group. None of the six infants had a recurrent feeding intolerance due to postoperative stricture or other gastrointestinal sequelae, and none of the infants developed postoperative NEC.

### When Should Aggressive Surgery Need to Be Considered?

In this study, six infants with NEC received aggressive surgeries without an absolute indication. These patients had a preoperative diagnosis of stage IIa or IIb but either had a recurrent worsening clinical presentation or had rapidly progressing deteriorated systematic symptoms despite repeated conservative treatment. The risk factors identified in this study that might be associated with the potential need for surgery included lower gestational age and birth weight, FGR, history of asphyxia, use of steroids and vascular active drugs before the onset of NEC, blood transfusion, and hsPDA. In addition, we also found that thrombocytopenia and increasing CRP were associated with worsening of the disease.

The findings in this study were consistent with a previous research, which suggested that deterioration of abdominal circumference over time, marked erythmia of the abdominal wall, fixed abdominal masses, and worsening systemic symptoms despite maximized medical therapy were all indications for surgical intervention (7). Therefore, we proposed that after maximum conservative treatment, if the patients had worsening abdominal symptoms accompanied by thrombocytopenia and increasing CRP, as well as previous high-risk factors such as FGR, history of asphyxia, use of steroids or vasoactive drugs before onset of NEC, and blood transfusion, an early aggressive surgical intervention should be considered. However, this hypothesis needs to be further verified in a larger controlled trial.

In the aggressive surgical group, intestinal perforation and peritonitis were identified during the operation in one case without signs of pneumoperitoneum in radiology. The other five cases had different degrees of intestinal necrosis, incomplete intestinal obstruction, gas accumulation in the intestinal wall, and ascites but did not develop perforation. Therefore, relying solely on radiology to decide the surgical indication seemed not enough, as the sensitivity to detect pneumoperitoneum and pneumatosis in the abdominal X-ray (AXR) was limited and depended on many confounders such as the experience of the radiologists and the timepoint when the AXR was performed. Nowadays, abdominal ultrasound (AUS) may represent an emerging method in evaluating the need for an operation in infants with Bell's stage II and stage III NEC (13). The ultrasound was more sensitive than AXR in identifying intra-abdominal fluid as well as the thickening of the bowel wall structures. The avascularity sign identified in AUS could indicate impending necrosis (14, 15). In this study, three out of the six aggressive surgical cases received pre-operative AUS. The AUS of case 3 indicated a thickened intestinal wall, fixed intestinal loop, and ascites, while the AXR only demonstrated a highly dilated intestinal tract with gas accumulation. Eventually, post-operative intestinal perforation and peritonitis were shown, which also confirmed the sensitivity of AUS.

Plenty of research had focused on finding promising predictors to identify infants with NEC who would ultimately require a surgical intervention. Tepas et al. (16) proposed a predictive model consisting of seven variables (MD7 parameters; positive blood culture, acidosis, bandemia, thrombocytopenia, hyponatremia, hypotension, and neutropenia) in their study and suggested that patients with at least three variables were more likely to progress to advanced disease and eventually for surgical intervention. Others had allocated clinical or radiological findings such as increased abdominal circumference over time, marked erythmia of the abdominal wall, and fixed abdominal masses as indications for infants who need surgery (7). Important clinical factors also included worsening apnea and bradycardia, temperature instability, and hypotension despite maximized medical therapy (7). In addition, some biomarkers such as fecal calprotectin, S100A12, IL8, and I-FABP were proposed to predict disease progression and surgical need in NEC (17–19). Recent advances in AUS also facilitated the identification of surgical needs in NEC (20). The validity of each single module in identifying potential surgical needs in patients with NEC needed to be further evaluated.

## Conclusion

In conclusion, our data suggested that aggressive operation before the identification of pneumoperitoneum in the radiology may improve the clinical outcomes in infants with severe necrotizing enterocolitis. Further studies are needed to verify our hypothesis and to better identify the requirements of early surgical intervention in NEC.

## Data Availability Statement

The raw data supporting the conclusions of this article will be made available by the authors, without undue reservation.

## Ethics Statement

This study was approved by the ethics committee of Tongji Hospital. Written informed consent from the participants' legal guardian/next of kin was not required to participate in this study in accordance with the national legislation and the institutional requirements.

## Author Contributions

XH conceived and designed the study. YW and YZ had roles in data collection and statistical analysis. YW was also responsible for writing and revising the article. XL, LC, and XH were responsible for review and revision. All authors reviewed and revised the manuscript and approved the final version.

## Conflict of Interest

The authors declare that the research was conducted in the absence of any commercial or financial relationships that could be construed as a potential conflict of interest.

## Publisher's Note

All claims expressed in this article are solely those of the authors and do not necessarily represent those of their affiliated organizations, or those of the publisher, the editors and the reviewers. Any product that may be evaluated in this article, or claim that may be made by its manufacturer, is not guaranteed or endorsed by the publisher.

## Abbreviations

AXR: abdominal X-ray
AUS: abdominal ultrasound
CBC: complete blood count
CRP: C-reactive protein
EBM: expressed breast milk
ELBW: extremely low birth weight
FGR: fetal growth restriction
hsPDA: hemodynamic significant patent ductus arteriosus
NEC: necrotizing enterocolitis
NRP: neonatal resuscitation program
PN: parenteral nutrition
PNAC: parenteral nutrition-associated cholestasis.
